# Long Noncoding RNA Hotair Promotes the Progression and Immune Escape in Laryngeal Squamous Cell Carcinoma through MicroRNA-30a/GRP78/PD-L1 Axis

**DOI:** 10.1155/2022/5141426

**Published:** 2022-04-04

**Authors:** Xiaowei Yuan, Qinhua Shen, Wenxue Ma

**Affiliations:** ^1^Department of Otorhinolaryngology, People's Hospital of Dongxihu District, Wuhan, China; ^2^Department of Pharmacy, People's Hospital of Dongxihu District, Wuhan, China; ^3^Department of Otorhinolaryngology-Head and Neck Surgery, Second People's Hospital of Jingmen, 39 Xiangshan Road, Jingmen Hubei Province 448000, China

## Abstract

Homeobox (HOX) transcript antisense RNA (Hotair) is elevated in many cancers significantly. However, the oncogenic role of Hotair in human laryngeal squamous cell carcinoma (LSCC) is still unknown. Thus, we explored the expression profile of Hotair and its function in LSCC. We observed high expression levels of Hotair in six LSCC cell lines compared to the human nasopharyngeal epithelial cell line. Knockdown of Hotair inhibited proliferation and enhanced apoptosis of Tu212 and Hep-2 cell lines in vitro. Moreover, the overexpression of hsa-miR-30a-5p inhibited the expression of GRP78 and PD-L1, but Hotair overexpression in LSCC cells rescues both proteins. Furthermore, the impacts of hsa-miR-30a-5p upregulation on the apoptosis and proliferation of LSCC cells were rescued by overexpression of Hotair. Finally, we combined si-Hotair and a VEGF inhibitor to treat LSCC cells in vitro or in vivo and surprisingly observed a significant inhibition of LSCC growth. In summary, these results indicate that Hotair displays an oncogenic role in both malignancy and immune escape in LSCC related to hsa-miR-30a-5p/GRP78/PD-L1 signaling. Therefore, Hotair may be a potential target for treating LSCC.

## 1. Introduction

Laryngeal squamous cell carcinoma (LSCC) is one of the most malignant neoplasms in the head and neck. About 60% of LSCC patients are in the terminal stages (clinical stages III and IV) at diagnosis [1]. The prognosis of LSCC remains poor, although there is a considerable improvement in surgical and oncological treatments [2]. The 5-year survival rate has declined from 66 to 63% during the past 40 years in LSCC [3]. This unsatisfactory curative ratio is primarily relevant to its unclear mechanism of oncogenicity. Thus, a better comprehension of the mechanisms in LSCC oncogenicity is necessary for screening more accurate and predictive biomarkers and for developing more effective therapeutic strategies.

lncRNAs are a heterogeneous group of noncoding polyadenylated RNAs longer than 200 nucleotides [4] and are involved in many human diseases, including cancers [5]. In many types of human cancers, lncRNAs are aberrantly expressed. Many reports have proved that HOX transcript antisense intergenic RNA (Hotair) is continually elevated in different cancers, such as breast cancer [6], esophageal cancer [7], lung cancer [8], and gastric cancer [9]. Moreover, Hotair expression positively correlates with poor prognosis. Hotair is closely associated with many cellular physiological processes, such as cell apoptosis, proliferation, angiogenesis, and tumor cell metabolism [7, 10, 11]. Although Hotair has been reported as a prognostic marker for LSCC, the specific mechanism is still unclear.

Here, we explored that Hotair was greatly upregulated in LSCC cell lines compared to NP69, as a ceRNA to regulate the 78 kDa glucose-regulated protein (GRP78) expression by sponging hsa-miR-30a-5p and then upregulating the expression level of PD-L1 in LSCC. Thus, Hotair plays a promotive role in LSCC progression and oncogenicity. Our works provides the first evidence for the relationship among Hotair, hsa-miR-30a-5p, GRP78, and PD-L1, providing new ideas on the LSCC therapy.

## 2. Materials and Methods

### 2.1. Chemical and Antibodies

The VEGF inhibitor, V1, was gained from Sigma–Aldrich. Antibodies used for western blotting were shown as follows: PD-L1 (13684S, Cell Signaling Technology, 1 : 1000), GRP78 (NBP1–06274, Novus Biologicals, 1 : 1000), and GAPDH (10494–1-AP, Proteintech, 1 : 1000).

### 2.2. Cell Culture

NP69, a human nasopharyngeal epithelial cell line, was purchased from Sigma–Aldrich (USA). The human LSCC cell lines LSC-1, Hep-2, Tu177, Tu212, Tu686, and UM-SCC-10A were obtained from the Chinese Academy of Sciences (Shanghai, China). NP69 cells were cultured in keratinocyte serum-free medium (keratinocyte-SFM; Gibco). LSCC cells were cultured in Dulbecco's modified Eagle's medium containing 10% fetal bovine serum. All cell lines were maintained at 37°C in a humidified atmosphere with 5% CO_2_ and tested negative for mycoplasma contamination every 3 months.

### 2.3. Lentivirus, Plasmids, and Oligonucleotides

Anti-hsa-miR-30a-5p oligonucleotides and their control (anti-NC) were obtained from Sangon Biotech (Shanghai, China). hsa-miR-30a-5p mimic, mi-NC, si-Hotair, and si-NC were also generated by Sangon Biotech. The sequences of the above primers and oligonucleotides are shown in Supplement Table 1. The plasmid for overexpressing Hotair (pHotair) was obtained from Genscript (Nanjing, China) with the pCDNA3.1 backbone.

### 2.4. Cell Transfection

Cells were seeded into 24-well plates. The cell confluency was at 80% for plasmid transfection and 50% for siRNA transfection. Plasmids and siRNAs were individually or cotransfected into Hep-2 and Tu212 cells with Lipofectamine 3000 (Invitrogen, USA) according to the manufacturer's protocol. Cells were harvested for detection at 48 h after transfection.

### 2.5. Quantitative Real-Time PCR Analysis

Total RNAs were extracted through miRNeasy and miRNA cleanup kits (Qiagen, Valencia, CA). The abundance of mRNA and miRNA was measured by TaqMan quantitative real-time PCR (qRT-PCR) method. For quantifying miRNA, stem-loop method was used and the primers and probes were purchased from TaqMan™ MicroRNA Assay (Applied Biosystems, Shanghai). All the reagents for reverse transcriptase and qRT-PCR reactions were from Applied Biosystems. The comparative cycle threshold (Ct) method (2-*ΔΔ*Ct) was used to analyze relative RNA expression (GAPDH normalized for mRNA expression and U6 RNA normalized for miRNA).

### 2.6. Western Blot

RIPA buffer (Beyotime, China) was used to extract proteins from cells, and BSA method (Beyotime, China) was used to quantify proteins. Then, protein was separated by 4-20% sodium dodecyl sulfate polyacrylamide gel electrophoresis (SDS-PAGE) at 120 V for 90 min and then transferred into PVDF membranes (Millipore, Billerica, MA) at 300 mA for 90 min. The membranes were incubated with primary antibodies at 4°C overnight after blocking with PBS containing 5% skimmed milk. Subsequently, the membranes were incubated with secondary antibodies (HRP) (Sino Biological, 1 : 5000) for 2 h at room temperature and detected using an ECL substrate kit (Beyotime).

### 2.7. Cell Proliferation Assay (CCK8)

Cell Counting Kit 8 (CCK8, Beyotime, Shanghai, China) was used to analyze cell proliferation.

5 × 10^3^ cells were seeded into one well in 96-well plates and cultured for 24 h, 48 h, 72 h, 96 h, and 120 h. At each time point, 10% CCK8 solution was added into wells and incubated for 2 h. The absorbance was measured at 450 nm.

### 2.8. Colony Formation

Six hours after transfection, cells were plated into 6-well plates at 1000 cells/well and routinely grown for 5 days. Then, crystal violet solution was added into wells for stain after fixing with methanol. Images were taken immediately.

### 2.9. Fluorescence-Activated Cell Sorting (FACS) Analysis

Cell apoptosis was detected by FACS. Cells with the density of 2 × 10^5^/well were plated into 24-well plates and transfected with si-Hotair or si-NC. Then, cells were stained by FITC-labeled annexin V/propidium iodide (PI) apoptosis detection kit (Invitrogen) according to the manufacturer's protocol at 72 h after transfection and analyzed by flow cytometry.

### 2.10. Prediction of the Interaction between GRP78, miRNAs, and Hotair

The prediction of miRNA target was performed by TargetScan Release 7.1 (https://www.targetscan.org/vert_71/). The online bioinformatics tool DIANA TOOLS (http://diana.imis.athena-innovation.gr/DianaTools/index.php) was used to predict the interaction between Hotair and hsa-miR-30a-5p.

### 2.11. RNA Immunoprecipitation Assay

EZ-Magna RIP RNA-Binding Protein Immunoprecipitation Kit (Millipore) was used to perform RNA immunoprecipitation (RIP) assays. Cells were lysed with RIP lysis buffer (Beyotime). Subsequently, lysates were incubated with magnetic beads conjugated to human anti-Ago2 antibody (66720-1-Ig, Proteintech, 1 : 200) or control IgG (A0408, Beyotime, 1 : 200) (Invitrogen) in RIP buffer. Then, coprecipitated RNAs were analyzed by qPCR.

### 2.12. RNA Pull-Down Assay

For the RNA pull-down assay, biotin-labeled Hotair probes, GRP78 probes, and control probes were designed and synthesized from GenePharma. Cell lysates were generated by RIP lysis buffer (Beyotime) from 2 × 10^7^ cells and incubated with probe overnight at 37°C. Then, C1 streptavidin magnetic beads (Invitrogen) were added and incubated for 2 hours at 25°C. The coprecipitated RNAs were analyzed by qPCR.

### 2.13. Luciferase Reporter Assay

The wild-type target fragment of hsa-miR-30a-5p in GRP78 and Hotair (WT) and their mutants (Mut) were cloned into the gGL3-promoter vector (Promega) at the HindIII and SpeI sites. Cells were cotransfected with luciferase plasmids and hsa-miR-30a-5p mimic or pHotair. Luciferase activities were analyzed using a dual luciferase reporter assay kit (Promega) on a luminometer (Lumat LB9507) at 48 h after transfection.

### 2.14. Lactate Dehydrogenase- (LDH-) Based T Cell Killing Assay

Peripheral blood mononuclear cells (PBMCs, Sailybio, Shanghai, China) were cultured in 1640/RPMI medium (Gibco, USA) containing 10% FBS (Gibco, USA). Twenty-four hours after miRNA or siRNA transfection, 1 × 10^4^ target cells (Hep-2 and Tu212) were seeded into triplicate in 96-well plates with effectors (PBMCs) at a ratio of 10 : 1 (*E* : *T*, effector : target). Mixed cells were cocultured in 1640/RPMI containing 5% FBS for 12 h at 37°C, 5% CO_2_. Then, cytotoxicity was monitored using the LDH release assay (Nonradioactive Cytotoxicity Assay, Promega). The absorbance was measured at 490 nm on a microplate reader (BioTek Instruments Inc., US). The cytotoxicity% was computed as the formula cytotoxicity% = (samples − *E* only blank)/(*T* only blank)∗100.

### 2.15. Enzyme-Linked Immunosorbent Assay (ELISA)

Supernatants from tumor cell and PBMC cocultures were collected to detect interleukin-2 (IL-2) and interferon-*γ* (IFN-*γ*) concentration, analyzed by ELISA kits (Sigma–Aldrich) according to the manufacturer's protocol using a Bio-Rad 550 microplate reader. Absorbance values were recorded with a standard curve, and the IFN-*γ* and IL-2 levels were calculated based on their absorbance.

### 2.16. Animal Model

Human CD34+ hematopoietic stem cell-engrafted NSG mice were obtained from the Jackson Laboratory (CD34+ hu-NSG™, Bar Harbor, ME, USA). All mice were maintained under specific pathogen-free conditions and adaptation for 2 weeks. 6 × 10^6^ Hep-2 cells were injected into the mice (*n* = 6) subcutaneously. Treatment started when tumor volume reached 80–150 mm^3^. Cholesterol-coupled si-NC or si-Hotair (GenePharma) combined with PBS or V1 were intratumorally injected into the tumor three times a week for two weeks. Tumors were measured using calipers every 3 d, and volumes (mm^3^) were calculated as follows: length × width^2^/2. 14 days after the last injection, mice were euthanized, and the tumors were carefully separated, followed by weighing and imaging.

### 2.17. Isolation of PBMC from the Mouse Blood

Blood (200 *μ*l) were harvested from tail veins at 14 days after the last injection using potassium EDTA-coated tubes. Red blood cells (RBCs) were lysed by lysis buffer (eBioscience) according to the manufacturer's instructions. Isolated PBMC were used for ELISA and T cell killing assays.

### 2.18. Statistical Methods

Values are reported as the mean ± SD indicated in each legend. Statistical comparisons among multiple groups were performed using one-way ANOVA followed by Tukey's multiple comparisons test. All other results were analyzed using two-tailed Student's *t*-test between two groups. Differences of *p* < 0.05, *p* < 0.01, and *p* < 0.005 were considered statistically significant. All statistical analyses were performed using GraphPad Prism 5 analytic software.

## 3. Results

### 3.1. Hotair Is Significantly Upregulated in LSCC

**Figure 1:**
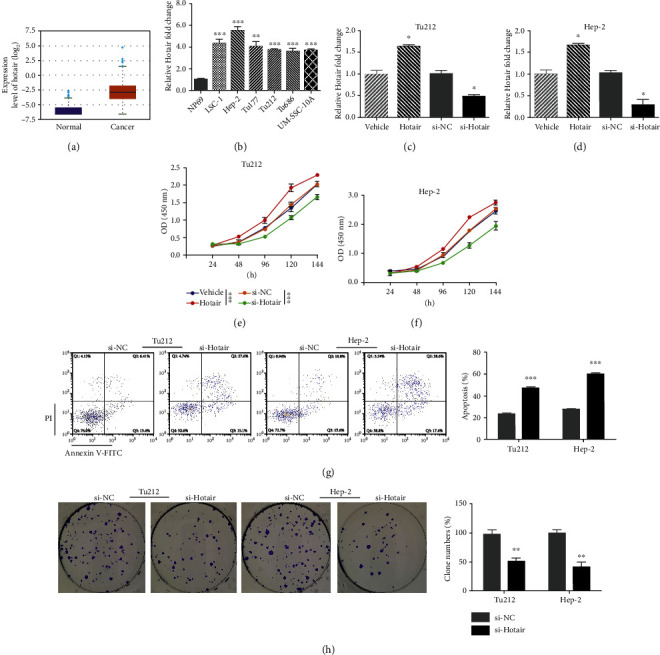
Hotair is frequently overexpressed in LSCC and mediates cell growth and apoptosis in LSCC cells. (a) The expression level of Hotair in HNSC tissues and normal tissues. (b) The relative expression of Hotair in six LSCC cell lines (LSC-1, Hep-2, Tu177, Tu212, Tu686, and UM-SSC-10A) and human nasopharyngeal epithelial cell line (NP69) analyzed by qRT-PCR. The relative expression of Hotair after Hotair overexpression and knockdown in (c) Tu212 and (d) Hep-2 cells analyzed by qRT-PCR. The cell viability after Hotair overexpression or knockdown in (e) Tu212 and (f) Hep-2 cells analyzed by CCK8. (g) The apoptosis after Hotair knockdown in Tu212 and Hep-2 cells. (h) The colony formation after Hotair knockdown in Tu212 and Hep-2 cells. Data are mean ± SD of at least three independent experiments; ^∗^*p* < 0.05; ^∗∗^*p* < 0.01; ^∗∗∗^*p* < 0.001.

According to clinical data from TCGA database, head and neck squamous cancer (HNSC) tumor tissues exhibit much higher expression of lncRNA Hotair than normal tissues (Figure 1(a)), indicating its potential important role in LSCC (a subtype cancer of HNSC). Next, we analyzed Hotair expression in six LSCC cell lines (Tu212, Hep-2, Tu177, Tu212, Tu686, and UM-SSC-10A) and one normal nasopharyngeal epithelial cell line (NP-9) using qPCR. The results showed that Hotair was significantly upregulated in LSCC (Figure 1(b)).

### 3.2. Hotair Promotes Proliferation and Inhibits Apoptosis of LSCC Cells In Vitro

To explore the role of Hotair in LSCC, we transfected pHotair or si-Hotair into Hep-2 and Tu212 cell lines and assessed cell viability and apoptosis. The results showed that Hotair expression was increased by pHotair and suppressed by si-Hotair (Figures 1(c) and 1(d)). In response to Hotair knockdown, a significant suppressive effect on cell proliferation was observed in Hep-2 and Tu212 cells, while the opposite effect was observed in Hotair-overexpressing cells (Figures 1(e) and 1(f)). The apoptosis results showed that apoptotic cells were higher in response to si-Hotair (Figure 1(g)). In contrast, si-Hotair-transfected LSCC cells generated far fewer colonies than the si-NC group (Figure 1(h)). These data demonstrate that Hotair boosts cell proliferation and reduces cell apoptosis in LSCC cells.

### 3.3. GRP78 Is a Functional Target of Hotair in LSCC Cells

**Figure 2:**
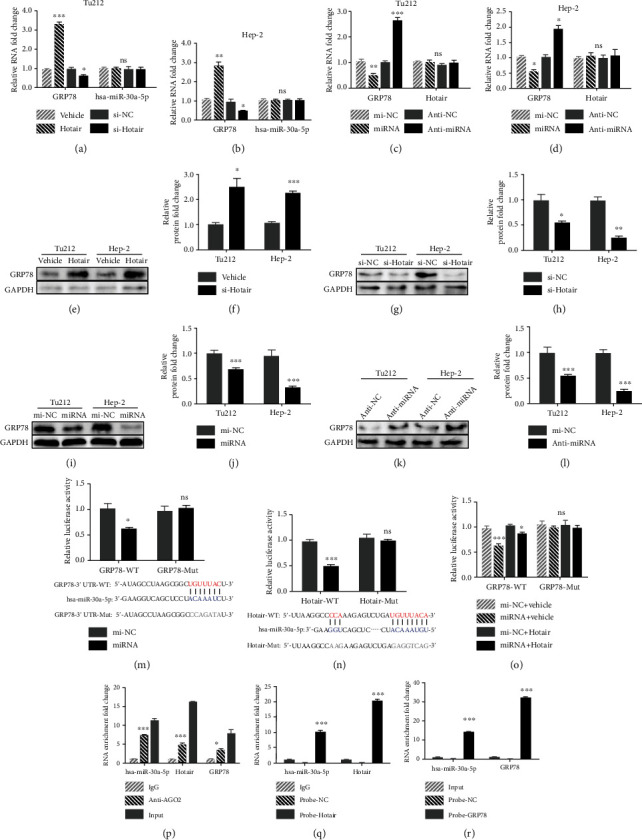
Hotair functions as a sponge for hsa-miR-30a-5p to increase GRP78 expression. The relative RNA expression of GRP78 and hsa-miR-30a-5p in (a) Tu212 and (b) Hep-2 cells 48 h after Hotair overexpression and knockdown as detected by qRT-PCR. The relative RNA expression of GRP78 and Hotair in (c) Tu212 and (d) Hep-2 cells 48 h after hsa-miR-30a-5p overexpression and knockdown as detected by qRT-PCR. Western blot analysis of GRP78 after Hotair (e) overexpression or (g) knockdown in Tu212 and Hep-2 cells. The relative expression level of GRP78 (f, h). Western blot analysis of GRP78 after hsa-miR-30a-5p (i) overexpression or (k) knockdown in Tu212 and Hep-2 cells. The relative expression level of GRP78 (j, l). (m) The luciferase reporter assay in Hep-2 cotransfected with control mi-NC or hsa-miR-30a-5p and wild-type or mutant target fragment of hsa-miR-30a-5p in GRP78 luciferase vector. The diagram showed the specific binding site between has-miR-30a-5p and GRP78-3′-UTR. (n) The luciferase reporter assay in Hep-2 cotransfected with control mi-NC or has-miR-30a-5p and wild-type or mutant target fragment of hsa-miR-30a-5p in Hotair luciferase vector. The diagram showed the specific binding site between Hotair and hsa-miR-30a-5p. (o) The luciferase reporter assay in Hep-2 cotransfected with control mi-NC or has-miR-30a-5p, vehicle or Hotair, and wild-type or mutant target fragment of hsa-miR-30a-5p in GRP78 luciferase vector. (p) RIP assay in Hep-2 cells using normal mouse IgG or human anti-Ago2 antibody, followed by qRT-PCR analysis for has-miR-30a-5p, Hotair, and GRP78 expression. (q) RNA pull-down assay in Hep-2 cells using control or Hotair probe, followed by qRT-PCR analysis for has-miR-30a-5p and Hotair expression. (r) RNA pull-down assay in Hep-2 cells using control or GRP78 probe, followed by qRT-PCR analysis for has-miR-30a-5p and GRP78 expression. Data are mean ± SD of at least three independent experiments; ^∗^*p* < 0.05; ^∗∗^*p* < 0.01; ^∗∗∗^*p* < 0.001.

Glucose-regulated protein 78 (GRP78) is associated with cancer development in many aspects, including tumor proliferation, angiogenesis, metastasis, and chemoresistance [12]. In human nasopharyngeal carcinoma, Hotair overexpression promotes tumor growth and angiogenesis by directly activating glucose-regulated protein 78 (GRP78) [13]. However, there is very little research regarding Hotair and GRP78 in tumors, not to mention LSCC. Therefore, we wanted to explore whether Hotair promotes LSCC through GRP78. Our data revealed that si-Hotair inhibited expression levels of GRP78 in Hep-2 and Tu212 cells, and Hotair overexpression promoted GRP78 expression at both the transcriptional (Figures 2(a) and 2(b)) and translational levels (Figures 2(e)–2(h)), suggesting that Hotair positively regulates GRP78 expression.

### 3.4. Hotair Serves as a Sponge for hsa-miR-30a-5p to Regulate GRP78

As mentioned above, Hotair upregulated the expression of GRP78, suggesting that there are mechanisms involved in between Hotair and GRP78. Given that lncRNAs sponge miRNAs and that miRNAs can reduce mRNA stability by targeting their 3′-UTR, we inferred that miRNAs might be involved in the regulation of GRP78 by Hotair. Using the online bioinformatics tool DIANA TOOLS (http://diana.imis.athena-innovation.gr/DianaTools/index.php), we found that there was a 10 nt interaction between Hotair and hsa-miR-30a-5p (Figure 2(m)). The 3′-UTR GRP78 mRNA was also predicted to be bound by hsa-miR-30a-5p using the biological prediction website (https://www.targetscan.org/vert_72/) TargetScan (Figure 2(n)). We then examined the expression of hsa-miR-30a-5p in Hotair knockdown and overexpressing LSCC cells and found that there was no difference in hsa-miR-30a-5p (Figures 2(a) and 2(b)). Furthermore, overexpression or inhibition of hsa-miR-30a-5p only inhibited or promoted mRNA and protein expression levels of GRP78, respectively, without any influence on Hotair expression (Figures 2(c), 2(d), and 2(i)–2(l)), which is a typical performance of sponge activity. We then confirmed the relationship among Hotair, hsa-miR-30a-5p, and GRP78 using a luciferase reporter assay. Overexpression of hsa-miR-30a-5p reduced luciferase activity of the GRP78 vector with the wild-type hsa-miR-30a-5p binding site, whereas it had no effect on the mutant vector (Figure 2(m)). On the other hand, high levels of hsa-miR-30a-5p also inhibited the luciferase activity of the Hotair vector containing the wild-type hsa-miR-30a-5p binding site, with no influence on the mutant vector (Figure 2(n)). Moreover, the hsa-miR-30a-5p-induced inhibitory effect on the GRP78 vector was rescued by overexpressing Hotair (Figure 2(o)), indicating the potential existence of sponge activity between Hotair and hsa-miR-30a-5p.

To further test whether these two factors interact with each other directly, we used RIP and RNA pull-down assays in Hep-2 cells. The RIP assay revealed that the anti-AGO2 antibody pulled down much more hsa-miR-30a-5p and Hotair than IgG (Figure 2(p)). Next, RNA pull-down assays were further used to identify the interaction between Hotair/hsa-miR-30a-5p/GRP78 using the biotin-labeled probes targeting Hotair and GRP78, respectively, in Hep-2 cells. As displayed in Figures 2(q) and 2(r), comparing to the control probe, both Hotair probe and GRP78 probe could pull down hsa-miR-30a-5p abundantly. Overall, these results demonstrate that Hotair regulates GRP78 mRNA by sponging hsa-miR-30a-5p in LSCC.

### 3.5. Hotair Promotes Cell Proliferation and Clone Formation through hsa-miR-30a-5p/GRP78

**Figure 3:**
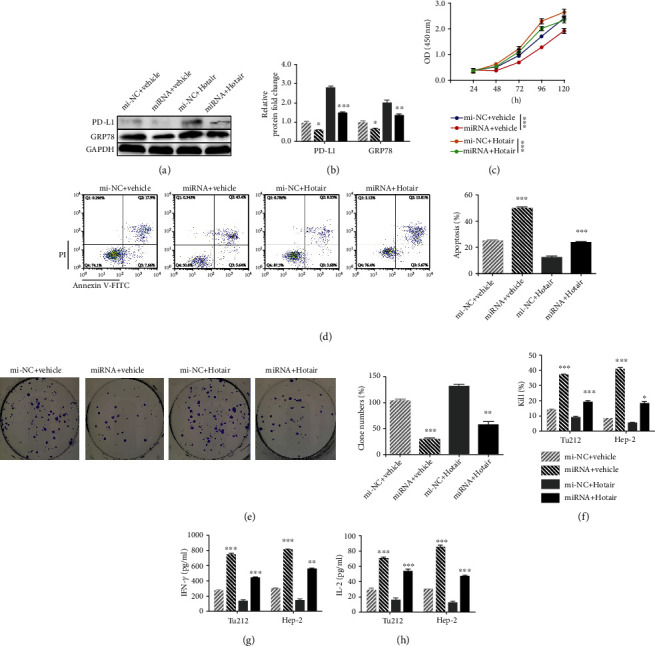
Hotair promotes the expression of GRP78 and PD-L1, mediates the proliferation, and helps to regulate the T cell-mediated immune responses in Tu212 and Hep-2 cells. Western blot analysis of GRP78 and PD-L1 after mi-NC or has-miR-30a-5p and vehicle or Hotair transfection in Hep-2 cells (a). The relative expression level of GRP78 and PD-L1 in Hep-2 cells (b). (c–e) The cell viability, apoptosis, and colony formation after mi-NC or has-miR-30a-5p and vehicle or Hotair transfection in Hep-2 cells. (f–h) T cell killing assay of target Tu212 and Hep-2 cells cocultured with PBMC, and ELISA detects the concentration of cytokines (g) IFN-*γ* and (h) IL-2. The targeted Tu212 and Hep-2 cells after 24 h transfected with mi-NC or has-miR-30a-5p and vehicle or Hotair were cocultured with PBMC, and the cytotoxicity was measured by the LDH release assay. Data are mean ± SD of at least three independent experiments; ^∗^*p* < 0.05; ^∗∗^*p* < 0.01; ^∗∗∗^*p* < 0.001.

GRP78 plays an important role in the antiapoptotic mechanisms of cancer cells [14]. Several studies have proven that GRP78 expression positively correlates with tumor development in renal cell carcinoma [15], lung cancer [16], and ovarian cancer [17]. Our data supported that Hotair upregulates GRP78 via hsa-miR-30a-5p, and downregulation of Hotair also inhibited cell proliferation and promoted apoptosis in LSCC. Therefore, we next explored the relationship between them. For that purpose, we created four groups of Hep-2 cells: cotransfected with mi-NC and vehicle, hsa-miR-30a-5p and vehicle, mi-NC and Hotair, or hsa-miR-30a-5p and Hotair. Interestingly, elevated GRP78 levels were observed in Hep-2 cells with Hotair overexpression compared to the vehicle group, regardless of whether mi-NC or hsa-miR-30a-5p was cotransfected (Figures 3(a) and 3(b)). GRP78 expression was also suppressed by hsa-miR-30a-5p relative to mi-NC, regardless of whether vehicle or Hotair was cotransfected (Figures 3(a) and 3(b)), consistent with previous results. However, the inhibition of GRP78 induced by hsa-miR-30a-5p was rescued by Hotair upregulation (Figures 3(a) and 3(b)). Subsequently, Hotair inhibited cell proliferation, whether cotransfected with mi-NC or hsa-miR-30a-5p (Figure 3(c)). Similarly, cell proliferation in Hep-2 cells treated with hsa-miR-30a-5p was promoted compared to that in the mi-NC group, whether vehicle or Hotair was cotransfected, indicating that hsa-miR-30a-p enhances cell proliferation (Figure 3(c)), while this promotion was repressed by Hotair (Figure 3(c)). Moreover, Hotair overexpression resulted in inhibition of apoptosis and elevated clone formation in Hep-2 cells, cells cotransfected with either mi-NC or hsa-miR-30a-5p (Figures 3(d) and 3(e)). After the overexpression of hsa-miR-30a-5p, apoptosis was promoted and clone formation was inhibited, in cells cotransfected with vehicle or Hotair (Figures 3(d) and 3(e)). However, this hsa-miR-30a-5p-induced change was rescued by Hotair overexpression (Figures 3(d) and 3(e)). Taken together, these results indicate that Hotair promotes cell viability and clone formation and inhibits apoptosis by upregulating GRP78 by hsa-miR-30a-5p-mediated sponging.

### 3.6. Hotair Regulates PD-L1 via hsa-miR-30a-5p/GRP78

Wang et al. reported that PD-L1 expression in LSCC was significantly higher than that in healthy tissues [18], and Kowalski et al. also confirmed that the higher the LSCC advancement was, the higher the expression of PD-L1 [19]. In triple-negative breast cancer, GRP78 interacts with PD-L1 in the ER region and increases PD-L1 levels by regulating its stability [20], but there is no research regarding GRP78/PD-L1 in LSCC. Therefore, we speculated that Hotair can finally regulate PD-L1 via hsa-miR-30a-5p/GRP78. Western blot analysis demonstrated that overexpression of Hotair markedly increased the PD-L1 level in Hep-2 cells (Figure 3(a)). Although overexpression of hsa-miR-30a-5p significantly inhibited PD-L1 levels, this effect was rescued by Hotair upregulation (Figure 3(a)). With our findings combined with the known regulation correction in GRP78/PD-L1, we suggest that Hotair and hsa-miR-30a-5p regulate PD-L1 expression via GRP78.

### 3.7. Hotair Helps LSCC Cells Negatively Regulate T Cell-Mediated Immune Responses

PD-L1 is involved in the escape of tumor cells from the host immune system [21]. Elevated expression of PD-L1 in tumor cells renders them less susceptible to specific T cell antigen receptor-mediated lysis by cytotoxic T cells in vitro [22]. Thus, we hypothesized that Hotair also functions as a PD-L1 expression enhancer that exacerbates immune escape in LSCC cells. A T cell killing assay was performed to evaluate the biological function of Hotair in Hep-2 and Tu212 cells. Our data demonstrated that overexpression of Hotair markedly suppressed T cell killing activity (Figure 3(f)). hsa-miR-30a-5p overexpression in LSCC cells activated the killing function of T cells, but overexpression of Hotair eliminated these effects (Figure 3(f)). Furthermore, we detected levels of IFN-*γ* and IL-2 in the medium using ELISA and identified a similar tendency in all groups (Figures 3(g) and 3(h)). Collectively, these results support the notion that Hotair facilitates tumor cell escape T cells via the hsa-miR-30a-5p/GRP78/PD-L1 pathway.

### 3.8. Combined si-Hotair and VEGF Inhibitor Display Better Antitumor Activity in LSCC Cells In Vitro

**Figure 4:**
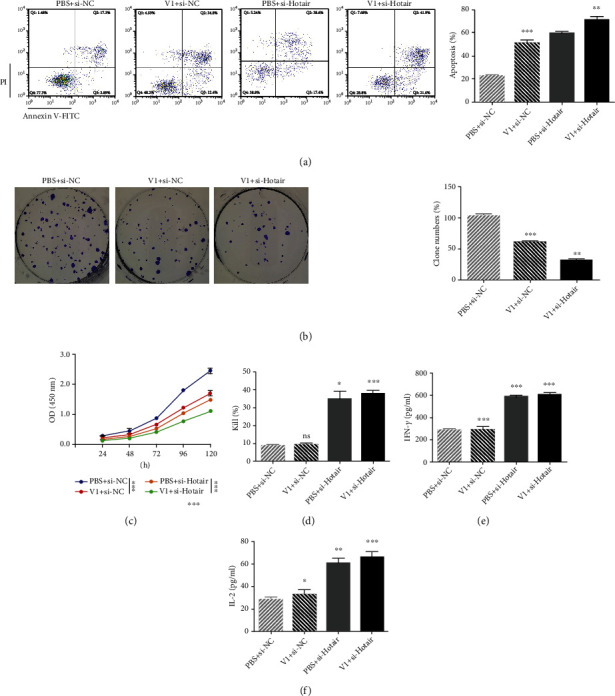
The combined si-Hotair and VEGF inhibitor show a better antitumor proliferation and anti-immune escape in vitro. The (a) apoptosis, (b) colony formation, and (c) viability of Hep-2 cells treated with PBS+si-NC, VEGF inhibitor V1+si-NC, PBS+si-Hotair, and VEGF inhibitor V1+si-Hotair, respectively. (d) T cell killing assay of each Hep-2 cell group cocultured with PBMC, and ELISA kit analyzed the concentration of cytokines (e) IFN-*γ* and (f) IL-2. Data are mean ± SD of at least three independent experiments; ^∗^*p* < 0.05; ^∗∗^*p* < 0.01; ^∗∗∗^*p* < 0.001.

There are multiple ongoing or completed randomized phase II and phase III studies of PD-1/PD-L1 antibodies combined with VEGF inhibitors [23], including hepatocellular cancer [24], renal cell carcinoma [25], and lung cancer [26]. Considering that combining VEGF inhibitors with PD-L1 antibodies obtains a better treatment effect, we wanted to explore whether si-Hotair and VEGF inhibitors display synergistic treatment effects in Hep-2 cells in vitro. We designed four groups of Hep-2 cell treatments: PBS and transfection with si-NC, V1 and si-NC, PBS and si-Hotair, or V1 and si-Hotair. Considering the antisurvival function of the VEGF inhibitor and si-Hotair in LSCC cells, we analyzed their synergistic effect on proliferation and apoptosis. Evaluation of apoptosis revealed the following tendencies: PBS+si-NC<V1+si-NC<PBS+si-Hotair<V1+si-Hotair (Figure 4(a)). Clone formation showed the tendency of PBS+si-NC>V1+si-NC> V1+si-Hotair (Figure 4(b)). Moreover, the proliferation of Hep-2 cells as analyzed by CCK8 assay exhibited a similar tendency to PBS+si-NC>V1+si-NC>PBS+si-Hotair>V1+si-Hotair. These in vitro results confirm that si-Hotair and VEGF inhibitor combination displays synergistic inhibition of LSCC cell proliferation.

In addition to the inhibition of proliferation ability, we evaluated the activity of this combination on immune escape. T cell killing results exhibited a much higher kill percentage in the si-Hotair treatment groups, while no significant improvement was observed in the V1 treatment group (Figure 4(d)). IFN-*γ* and IL-2 levels detected by ELISA in the supernatant of the T cell killing assay displayed a similar trend, with a slight difference in that V1 induced a slight improvement (Figures 4(e) and 4(f)). These data indicate that the combination of si-Hotair and VEGF inhibitor exerts better on anti-immune escape activity in LSCC cells.

Together, our findings demonstrated that combination of si-Hotair and the VEGF inhibitor V1 exhibits better anti-LSCC activity in vitro, both with respect to antitumor proliferation and anti-immune escape.

### 3.9. Combined si-Hotair and VEGF Inhibitor Reduce Tumorigenicity In Vivo

**Figure 5:**
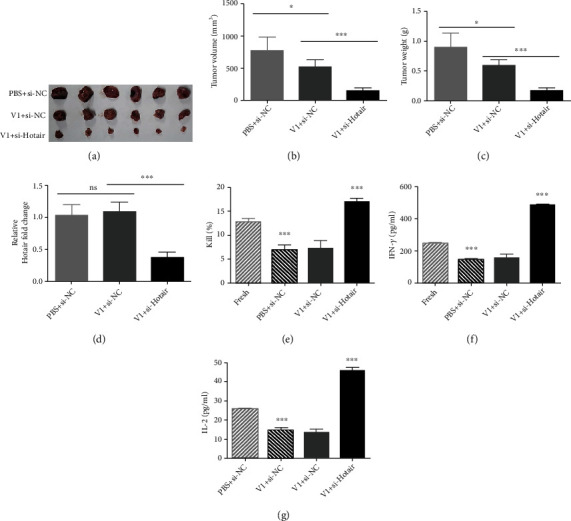
The combined si-Hotair and VEGF inhibitor show a better antitumor function in vivo. (a–d) The representative image showed subcutaneous tumors in CD34+ hu-NSG™ mice injected with PBS plus cholesterol-coupled si-NC, or V1 plus cholesterol-coupled si-NC, or V1 plus cholesterol-coupled si-Hotair. The (b) volume, (c) weight, and (d) expression levels of Hotair of subcutaneous tumors in each group were analyzed. (e) The T cell activity of PBMC cells in each group, and ELISA analyzed the concentration of cytokines (f) IFN-*γ* and (g) IL-2. PBMCs were collected from tail veins at two weeks after the last injection of cholesterol-coupled oligomers. Data are mean ± SD of at least three independent experiments; ^∗^*p* < 0.05; ^∗∗^*p* < 0.01; ^∗∗∗^*p* < 0.001.

Following the above observations, we further verified these in vitro findings using an in vivo xenograft model. Hep-2 cells were injected into the dorsal flank of CD34+ hu-NSG™ mice, and after two weeks, the subcutaneous tumors reached 80–150 mm^3^ in volume. Then, the mice were intratumorally injected with PBS plus cholesterol-coupled si-NC, V1 plus cholesterol-coupled si-NC, or V1 plus cholesterol-coupled si-Hotair three times weekly for two weeks. Comparing those groups, si-Hotair combined with V1 resulted in the greatest reduction in tumor growth, but V1 plus si-NC also inhibited tumor growth (Figures 5(a)–5(c)). In the other three groups, both tumor volumes and weights were distributed as follows: PBS plus si-NC>V1 plus si-NC>V1 plus si-Hotair (Figures 5(a)–5(c)). Hotair levels in tumors were analyzed to confirm Hotair knockdown (Figure 5(d)).

PD-L1 also plays an important role in various malignancies by attenuating the host immune response to tumor cells and exhausting T cells [27, 28]. The above data indicated that Hotair upregulates PD-L1 and that si-Hotair promotes T cell killing in vitro, so we further assessed whether si-Hotair attenuates the exhaustion of T cells induced by PD-L1 in vivo. We collected PBMCs from CD34+ hu-NSG™ mice at the end point and evaluated T cell activity using a T cell killing assay, including one group with only fresh PBMCs. Our results revealed that the group injected with both si-Hotair and V1 exhibited the highest levels of active T cells (Figure 5(e)), consistent with the in vitro results. T cell activity in the other three groups was distributed as follows: fresh PBMCs>V1 plus si-NC>PBS plus si-NC (Figure 5(e)), indicating that tumor-pretreated PBMCs exhibited exhaustion to some degree. Furthermore, the levels of IFN-*γ* and IL-2 showed a similar trend (Figure 5(f)).

Together, these in vivo results demonstrated that combination of si-Hotair and VEGF inhibitor exhibits strong anticancer function in LSCC tumors by inhibiting tumor growth and activating T cell-mediated immune responses.

## 4. Discussion

LSCC is one of the most malignant and frequent tumors in the head and neck, while clinical studies and satisfactory treatment strategies have not been achieved [29]. It is an emergency to identify specific molecular signatures. Hotair is a cancer-related lncRNA [6, 7, 30, 31] and found to promote the tumor development in many cancers [8, 10, 13]. Our findings showed that Hotair was especially upregulated in LSCC cells. Hotair knockdown inhibited cell proliferation and improved the apoptosis of LSCC cells. What is more, it suppressed colony formation in vitro, consistent with previous research [29, 32]. Additionally, Hotair is related to the immune escape of tumor cells, which is the first report to describe the new function of Hotair in cancer development. Therefore, our data highlight that Hotair promotes the proliferation and immune escape of LSCC cells.

The aberrant activity of Hotair can occur at different levels, such as regulating chromatin status and transcriptional silencing as a molecular scaffold or control gene expression by sponging to miRNAs [12]. hsa-miR-30a-5p acts as a negative regulator of tumor proliferation and migration [33–35] and has been reported to play a role in many diseases, such as gastric cancer [35], hepatocellular cancer [34], and renal cancer [33]. Furthermore, many lncRNAs have been reported to act as ceRNAs of hsa-miR-30a-5p [33–35], including Hotair [10]. However, Hotair, a ceRNA of hsa-miR-30a-5p, has only been reported in gastric cancer [10], and there are no reports on these effects in LSCC. Our research showed that Hotair acts as a ceRNA of hsa-miR-30a-5p in LSCC. Hotair directly binds to hsa-miR-30a-5p and inhibits the interaction between hsa-miR-30a-5p and GRP78. Zhang et al. studied Hotair in the context of gastric cancer and found that Hotair is negatively correlated with hsa-miR-30a-5p, which is contradictory to our results [10].

GRP78 is an immunoglobulin heavy chain-binding protein (BIP) that belongs to the heat shock protein 70 (HSP70) family [36]. In cancer cells, GRP78 contributes to cancer progression via maintaining cellular homeostasis through responding to the unfolded protein response [37]. GRP78 overexpression has been observed and usually involved in tumor malignancy or poor prognosis [38, 39]. Chen et al. identified the existence of a targeted relationship between hsa-miR-30a-5p and GRP78 in cardiac muscle and vascular smooth muscle cells [40]. Coincidentally, GRP78 was also identified as a functional target of Hotair and is involved in Hotair-mediated angiogenesis in nasopharyngeal carcinoma with no reports of any microRNA involvement [13]. However, the exact mechanism is still not clear. According to previous works and considering the heterogeneity in different cancers, we hypothesized that GRP78 expression could be controlled by Hotair by sponging miR30a in LSCC cells. As a result, we found that hsa-miR-30a-5p binds to the 3′-UTR of GRP78 mRNA and that Hotair functions as a hsa-miR-30a-5p sponge to promote GRP78 expression.

Several reports have characterized the role of GRP78 on PD-L1 levels through posttranslational modification and on prognosis in triple-negative breast cancer [20, 41]. Therefore, we think it is necessary to identify the relationship between PD-L1 and Hotair or hsa-miR-30a-5p to deeply investigate the role of Hotair in LSCC. Fortunately, we explored the function of Hotair in PD-L1 regulation among LSCC, identifying Hotair an ideal target. Once Hotair was knocked down, tumor growth stopped or was inhibited by suppressing tumor proliferation and improving the T cell-induced immune response simultaneously.

VEGF inhibitors combined with PD-L1 inhibitors are under investigation in clinical trials. VEGF is reduced in responders and increased in nonresponders after therapy with anti-PD-L1 in metastatic melanoma patients [42]. In mouse models, combination of anti-PD-1/PD-L1 with VEGFR-2 inhibitor displays synergistic effects on inhibiting tumor growth [26, 43]. Other reports states that angiogenesis represses T cell infiltration through different processes and that the combination therapy benefits T cell infiltration by increasing the number of high endothelial venules [44]. Previous studies demonstrated that VEGF enhances the invasion of Hep-2 and LSCC cell lines [45]. Our data support that the combination of si-Hotair and a VEGF inhibitor more strongly inhibits proliferation. Moreover, the integration of si-Hotair and VEGF also activated T cells through hsa-miR-30a-5p/GRP78/PD-L1. As a result, our in vivo data revealed the greatest tumor inhibition activity for the combination of si-Hotair and VEGF inhibition, which was attributed to its dual antitumor functions. Thus, si-Hotair and VEGF inhibitors represent a potential treatment for clinical LSCC patients.

Small interfering RNA (siRNA) presents remarkable ability due to its silence function to downregulate the expression of disease-relevant genes [46]. However, siRNAs are large macromolecules negatively charged, which results in the difficulty to cross cell membranes. On the other hand, they are also easily depredated by plasma enzymes or hepatic/renal clearance sequestration [46]. These problems severely limit siRNAs' utility in therapy. In our study, we chose to inject si-Hotair directly into tumors in mice. However, almost all the current siRNA delivery system in clinic is based on liposomes, polymeric and inorganic nanoparticles, aptamers, and chemical modification of siRNAs, which showed a well-delivered efficiency in the liver. Importantly, Craparo et al. developed a novel protonable copolymer based on polyaspartamide which could form an electrostatic complex with siRNA and be capable for the inhalation route [47]. We think this system may be useful for delivering our si-Hotair to treat LSCC. si-Hotair inhibits the growth of tumors through direct injection, but we may consider other new drug delivery methods in the future.

## 5. Conclusions

**Figure 6:**
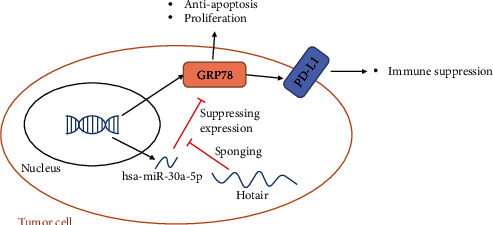
The scheme for the pathway in Hotair/has-miR-30a-5p/GRP78/PD-L1 in tumor cells.

As showed in Figure 6, our findings clearly indicate that Hotair functions as a positive regulator of LSCC through posttranscriptional alteration of GRP78 stability via coordinated regulation of hsa-miR-30a-5p. Furthermore, Hotair promotes immune escape by upregulating the expression of PD-L1 through hsa-miR-30a-5p/GRP78. Combining si-Hotair and VEGF inhibitor greatly reduces tumor growth both in vitro and in vivo. Therefore, Hotair may represent a promising druggable target in LSCC patients.

## Data Availability

The data used to support the findings of this study are available from the corresponding author upon request.

## Supplementary Materials

Supplementary Materials Table S1: primer sequence for qPCR.
