# Supplementary material for: Long Noncoding RNA Hotair Promotes the Progression and Immune Escape in Laryngeal Squamous Cell Carcinoma through MicroRNA-30a/GRP78/PD-L1 Axis
Source: J Immunol Res. 2022 Apr 4;2022:5141426. doi: 10.1155/2022/5141426 (PMC9001128; doi:10.1155/2022/5141426)
Supplement: Supplementary Materials — Table S1: primer sequence for qPCR. [file 5141426.f1.docx]

**Table S1: Primer sequence for qPCR**

| Primer name | Sequence |
| --- | --- |
| Hotair-qPCR-F | 5′-ACGGAACCCATGGACTCATA-3′ |
| Hotair-qPCR-R | 5′-TTGGGGAAGCATTTTCTGAC-3′ |
| GAPDH-qPCR-F | 5′CATGTTCGTCATGGGTGTGAA-3′ |
| GAPDH-qPCR-R | 5′GGCATGGACTGTGGTCATGAG-3′ |
| GRP78-qPCR-F | 5'-GATTGGACAAGAGAGAGGGTGA-3' |
| GRP78-qPCR-R | 5'-CCATAACACGCTGGTCAAAGTC-3' |
| PD-L1-qPCR-F | 5’-TGTACCACGTCTCCCACATAACAG-3’ |
| PD-L1-qPCR-R | 5’-ACCCCACGATGAGGAACAAA-3’ |
